# The Multi-Loci Genotypes of the Myostatin Gene Associated with Growth Indicators of Intensively Fattened Lambs of Latvian Sheep

**DOI:** 10.3390/ani14213143

**Published:** 2024-11-01

**Authors:** Ilva Trapina, Daina Kairisa, Samanta Plavina, Nikole Krasnevska, Jegors Paramonovs, Liga Senfelde, Natalia Paramonova

**Affiliations:** 1 Genomics and Bioinformatics, Institute of Biology, Faculty of Medicine and Life Sciences, The University of Latvia, Jelgava Str. 3, LV-1004 Riga, Latvia; samanta.plavina@lu.lv (S.P.); krasnevska@gmail.com (N.K.); jegors.paramonovs@lu.lv (J.P.); natalia.paramonova@lu.lv (N.P.); 2 Institute of Agrobiotechnology, Faculty of Agriculture, Latvian University of Life Sciences and Technologies, Liela Street 2, LV-3001 Jelgava, Latvia; daina.kairisa@lbtu.lv (D.K.); shenfeldel@gmail.com (L.S.)

**Keywords:** intensive fattening, feed efficiency, myostatin, Latvian sheep, multi-loci genotype, genetic markers

## Abstract

Myostatin (MSTN), a member of the TGFβ superfamily, negatively affects skeletal muscle growth and controls muscle development at stages of pre-natal muscle growth. Marker-assisted selection with molecular markers of the MSTN gene can be used in animal husbandry for higher-quality and more economically profitable lambs. The study aims to identify multi-locus genotypes within the MSTN gene regions as molecular markers for fattening, feed efficiency, and carcass traits in Latvian sheep. This analysis was conducted on 76 intensively fattened male lambs representing six breeds in Latvia. Nine genotypes were identified based on four SNPs in the promoter and exon 1 regions, while six and four SNPs in introns 1 and 2 led to the formation of 14 and 10 genotypes, respectively. Six genotypes identified in the 3′UTR were significantly linked to traits such as birth weight, slaughter yield, muscle development, fat depth, average daily gain, dry matter intake, and feed efficiency. Genotypes of intron 1 were associated with slaughter yield, while genotypes of intron 2 were linked to fat depth, dry matter intake, and residual weight gain. The study concludes that MSTN gene SNPs can be used in selection to enhance sheep breeds in Latvia and improve economic outcomes for breeders.

## Simple Summary

Myostatin (MSTN), a gene of the TGFβ superfamily, can be used in sheep husbandry to improve lamb quality and profitability. This study aims to identify multi-locus genotypes within the MSTN gene regions as molecular markers for fattening, feed efficiency, and carcass traits in breeds in Latvia. The analysis was conducted on 76 intensively fattened lambs from six breeds in Latvia. Nine genotypes were identified based on four SNPs in the promoter and exon 1 regions, while six and four SNPs in introns 1 and 2 led to the formation of 14 and 10 genotypes, respectively. Genotypes of intron 1 were correlated with the slaughter weight percentage, whereas genotypes of intron 2 were associated with fat depth, dry matter intake, and residual weight gain. Six genotypes in the 3′UTR were linked to crucial traits such as birth weight, slaughter weight percentage, muscle development, fat depth, average daily gain, dry matter intake, and feed efficiency. The research finds that MSTN gene SNPs may be utilised in the selection process to strengthen sheep breeds in Latvia.

## 1. Introduction

Myostatin (MSTN), a member of the TGFβ superfamily, negatively impacts skeletal muscle growth [1,2,3] by regulating muscle development during key phases of pre-natal growth, including muscle precursor proliferation, myoblast proliferation, and differentiation [4]. Downregulation of MSTN gene expression leads to increased muscle cell numbers (hyperplasia) and sizes (hypertrophy) [5].

Genetic mutations in the MSTN gene lead to a “double muscle” trait, which is crucial for enhancing animal meat production and providing high-quality protein for human consumption [6,7]. Over the past twenty years, MSTN gene genotyping across various breeds has revealed not only the presence of specific polymorphisms but also the variability of these polymorphisms among different breeds [1,4,8,9,10,11,12,13,14].

The Ensemble database (info 15 October 2024) contains information about 67 polymorphisms in the region from the beginning of the 5′UTR to the 3′UTR of the sheep MSTN gene. However, only eight are located in exons: three in exon 1, two in exon 2, and three in exon 3. Of the MSTN gene coding variations mentioned in the database, only two SNPs are found in scientific publications: rs417816017 (c.101A>G) in exon 1 [15] and rs868996529 (c.960del G) in exon 3 [16].

In 2018, Trukhachev reported only three variations in exons [17]. Also, publications contain information that cannot be found in the database about the following variations in exon 3: c.1009G>C, which has been found in Nilagiri sheep [18], and c.782_783insT and c.940G>T in Stavropol Merino sheep [17]. In summarising the information in the publications and the database—11 SNPs have been detected in the coding part of the MSTN gene so far.

Of all the SNPs located in the MSTN gene exon regions, seven (two in exon 1 and five in exon 3) affect the amino acid chain of the MSTN protein. In addition, four of the five variations located in exon 3 result in the formation of a stop codon [15,17].

Looking at the information about the variation in the coding region in the database, it can be seen that there is no information about the distribution of genotypes in different populations/breeds for four, including rs868996529; in the case of the other four, including rs417816017, the variation has been found in one or two breeds, but rs410961001 (c.384C>G), localised in exon 2, has been found in seven breeds. Additionally, the existence of the variations rs417816017 [15] and rs868996529 [16], as well as c.1009G>C [18], c.782_783insT, and c.940G>T [17], have been described each in one sheep breed.

The available information confirms the previously expressed hypothesis [17] about the conservation of the coding part of the MSTN gene and the importance of polymorphisms in the 5′UTR, introns and 3′UTR part.

Multi-loci genotypes in the intron 1 region of the MSTN gene have been associated with various traits in different breeds [12,13,19]. However, these associations have not been consistently replicated in other breeds. In a single-locus analysis, the frameshift mutation c.960delG in the MSTN gene of white sheep in Norway has been linked to reduced fat accumulation and increased muscle mass [16,20]. Another SNP, c.*1232G>A (rs408469734), located in the 3′UTR of the MSTN gene, creates a microRNA binding site [8], leading to a significant reduction of about two-thirds of MSTN protein in the bloodstream and resulting in increased muscularity [20]. This SNP is also associated with various phenotypic traits [13,14,21,22,23].

Recent research suggests that genetic variations, such as MSTN gene polymorphisms, vary among breeds [3,7], highlighting the importance of breed-specific studies. In Latvia, there is one native breed (around 70% from pure-breeds)—the Latvian dark-head (Latvijas tumšgalve; LT)—and another nine breeds with established breeding programmes [24]. Latvian dark-head is a mother breed with high reproducibility, but breed selection aims to improve the breed’s meat productivity.

According to Eurostat data [25,26], Latvia’s sheep population is declining, but the demand for high-quality sheep meat is rising. Improving the development, productivity, and reproductive characteristics of sheep breeds in Latvia is crucial to meeting the growing demand for meat, preventing further population loss, and addressing feed costs and methane emissions challenges.

Selective breeding of animals with enhanced feed efficiency effectively lowers feeding costs and reduces methane emissions [27,28,29,30,31], improving fattening and carcass traits. Marker-assisted selection (MAS) is an effective approach for identifying the most superior lambs for breeding [2,18,32].

There are limited data on the associations between certain genetic loci and important traits in the Latvian sheep population [14,33,34]. This study provides an opportunity to investigate genetic variations linked to weight gain, feed efficiency, and carcass characteristics in Latvian sheep raised under controlled, intense, fattening settings. This study also aims to identify multi-loci genotypes within the MSTN gene regions as molecular markers for fattening, feed efficiency, and carcass indicators of sheep in Latvia. Applying scientifically established correlations between weight gain, feed efficiency, carcass indicators, and genetic markers in a particular area can be employed in marker-assisted selection as a cost-effective and efficient method to improve breeds and enhance feed efficiency. This approach saves breeders significant time and costs in the breeding process.

## 2. Materials and Methods

### 2.1. Fattening Characteristics of Intensively Fed Lambs

Complete details on the lambs, feeding conditions, diet during intensive fattening, phenotypic measurements, and indicator calculations have been previously published in our studies [14,31,34,35].

In accordance with the requirements of the breeding programme [36], 76 purebred male lambs from six breeds in Latvia (Latvian dark-head (Latvijas tumšgalve; LT), Merinolandschaf (MLS/Württemberger), Île de France (IF), Charollais (CH), Dorper (DOR), and Texel (TEX)) were intensively fattened during the summer of 2022 at the “Klimpas” ram breeding control station in collaboration with the Latvian Sheep Breeders Association [14,31,34,35]. The lambs were fed according to an intensive fattening control protocol with combined, concentrated feed supplemented with hay, mineral feed, salt licks, and unlimited water from automatic waterers [37]. During the study, all animal care adhered to animal welfare requirements.

Before and after fattening, the lambs were weighed on an electronic scale with an accuracy of 0.01 kg and measured using Mindray Dp-50 Vet ultrasound (US) equipment along the longest back muscle (*Longissimus dorsi*). Each lamb’s average daily gain (ADG) was calculated based on changes in body weight adjusted for the lambs’ ages at 90 and 150 days over the 60-day intensive fattening period. Additionally, based on US measurements and gained body weight, the change (delta) of muscle depth (ΔMD) and fat thickness (ΔFT) of the 13th rib per one kg gained body weight were calculated.

The calculated ADG, dry matter intake (DMI), and live weight values were used to determine several efficiency metrics (feed efficiency (FE), feed conversion ratio (FCR), relative growth rate (RGR), and Kleiber’s ratio (KR)) and residual indicators (residual feed intake (RFI), residual weight gain (RWG), and residual intake and body weight gain (RIG)). These calculations were based on previously published formulas [27,28,31].

The EUROP classification system was used to evaluate carcass conformation and fatness [38]. This system categorises carcass or muscle development into five classes: E (1)—excellent, U (2)—very good, R (3)—good, O (4)—average, P (5)—poor. Fat stratification is also classified into five levels, ranging from 1 (very lean) to 5 (very fat). The visual assessment of the lamb carcasses, determining both conformation and fatness, was conducted by two independent assessors.

The slaughter yield was calculated by determining the proportion of meat obtained at slaughter relative to each lamb’s live weight at the processing time.

### 2.2. DNA Extraction

Blood samples were collected from the lamb’s jugular vein to extract genomic DNA using a DNA extraction kit from Fermentas (part of Thermo Fisher Scientific, Waltham, MA, USA). DNA quality and quantity were assessed using agarose gel electrophoresis and spectrophotometry techniques.

### 2.3. MSTN Gene Multi-Loci Genotypes

The complete sequencing of the MSTN gene was conducted using the Illumina MiSeq DNA sequencing system (Illumina, San Diego, CA, USA). Clean reads were aligned to the sheep reference genome (GCF_016772045.1_ARS-UI_Ramb_v2.0, NCBI) and variable loci were identified using Geneious Prime^®^ 2023.2.1 (https://www.geneious.com; accessed on 17 February 2023). Details on the analysis and distributions of variable loci of sheep breeds in Latvia have been published [14].

Single-locus genotypes and allele frequencies were calculated by direct counting and have been published previously [14]. These data were used to create multi-loci genotypes created by splitting the MSTN gene into regions: promoter and 1st exon, 1st intron, 2nd intron, and 3′UTR. Genotypes with five or fewer occurrences were grouped as rare genotypes to simplify the association analysis. The multi-locus genotypes are presented using standard IUB/IUPAC nucleic acid codes, representing the genotype of two alleles at a single locus with a single letter.

All haplotypes of the collection samples were generated using the software DnaSP6.12.03 [39].

### 2.4. Statistical Analyses

General and analytical statistics were conducted with SPSS v.25 [40]. The mean with standard deviation (SD) and median with interquartile range (IQR) for the indicators of each multi-locus genotype group were calculated from the measurement data. Depending on the normality of the data and/or the homogeneity of variances, appropriate statistical tests (*t*-tests, ANOVA, Kruskal–Wallis, or Median test) were applied to assess the differences between genotype group data in all 76 samples and within the 48 LT sample group. A significance level of *p* < 0.05 was used to define statistically significant results.

## 3. Results

A total of 23 SNPs with variability in at least one sample were detected across all 76 lamb samples [14]. All variable SNPs in the MSTN gene were constructed by combining 24 distinct multi-loci genotypes. Reducing the number of loci to 16 by excluding those that were variable in only one sample or who were in complete linkage disequilibrium did not decrease the number of genotypes [14]. Among these 16 loci, 12 multi-locus genotypes were found in only one sample.

Sixteen unique haplotypes were constructed, incorporating all 23 SNPs (Table S1). When excluding SNPs that were in linkage disequilibrium or detected in only one sample, 15 haplotypes were identified. Of these, eight can be considered frequent (occurring more than five times), while seven were detected once or twice. The most common haplotype, with common alleles at all loci, was found in 44.47% of the overall group and 51.04% of the LT group.

To streamline the association analysis, the MSTN gene was divided into four regions: the promoter and exon 1, intron 1, intron 2, and the 3′UTR. This division helped reduce the number of multi-locus genotypes.

### 3.1. Genotypes of Promoter and Exon 1 Region of MSTN Gene

The promoter region includes SNPs rs411139795 (c.-40C>A) and rs119102824 (c.-37T>C), while exon 1 contains the SNPs New SNP (c.84G>A) and rs417816017 (c.101A>G). The last two SNPs were variable only in Merinolandschaf (MLS) samples (Table S1). No statistically significant associations were found between these SNPs and any growth traits in single-locus analyses (Table S2) [14].

**Figure 1:**
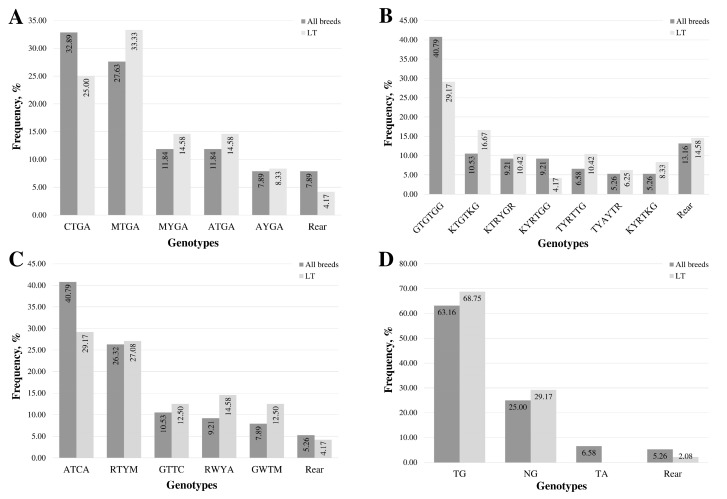
Distribution of multi-locus genotypes in the MSTN gene regions: (**A**) promoter and exon 1, (**B**) intron 1, (**C**) intron 2, and (**D**) 3′UTR. “Rear” denotes genotypes with fewer than five samples. The sequence of multi-locus genotypes is represented using standard IUB/IUPAC nucleic acid codes.

The most frequent genotype in the promoter and exon 1 region is CTGA, found in 36.84% of samples (Figure 1A). The second most frequent genotype, MTGA, was detected in 27.63% of the samples and was the predominant form in 33.33% of the LT samples.

The associations between promoter and exon 1 region genotypes, muscle evaluation for all samples, and slaughter yield for LT samples were on the borderline of statistical significance but not significant (*p* = 0.05–0.09).

### 3.2. Genotypes in the Intron 1 Region of the MSTN Gene

Nine SNPs were identified in the intron 1 region, with seven being clustered within a 400 bp region [14]. One SNP, rs417602601(c.373+241T>C), was found only in heterozygous form in the CH breed lamb (Table S1). Also, rs119102826 (c.373+241T>C), rs408710650 (c.373+563G>A), rs419902890 (c.373+607G>A), and rs399737483 (c.374−54C>T) are in complete LD with each other [14].

In all samples, the most frequent multi-locus genotype, detected in 40.79% of cases, was GTGTGG, consisting of homozygous forms of common alleles at all loci (Figure 1B). The proportion of other frequent genotypes (occurring in more than five samples) was 10% or less. In LT lambs, the most frequent multi-locus genotype, consisting of homozygotes for common alleles at all loci, was identified in 29.17% of the lambs. The KTGTKG genotype, featuring two heterozygous loci, was found in 16.67% of the lamb cohort.

A statistically significant association between intron 1 region genotypes and slaughter yield was found in the all-breed lambs’ (*p* = 4.48 × 10^−2^) and LT (*p* = 2.29 × 10^−2^) groups.

In the single-locus analysis (Table S2), the association with slaughter yield was identified with c.373+259G>T (rs119102828) SNP. Lambs with the most common GG genotype had a slaughter yield of 45.72 ± 2.53%, while those with the heterozygous GT genotype had a lower percentage of 43.79 ± 2.38%. Within the LT group, the Ms7 SNP showed an association with slaughter yield, where heterozygous GT samples had the highest value at 44.54 ± 2.27% and homozygous TT samples, carrying the rare allele, had the lowest value at 42.75 ± 1.40%.

**Figure 2:**
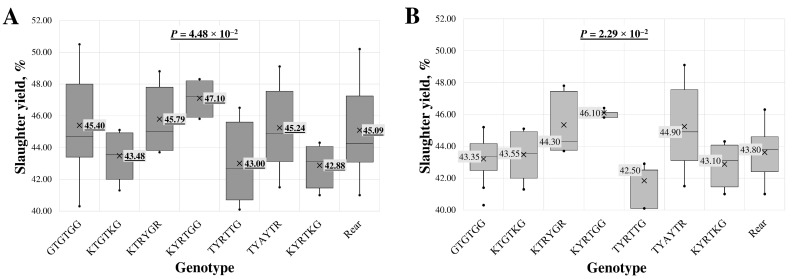
Box plot of slaughter yield for intron 1 region multi-locus genotypes in (**A**) all-breed lambs and (**B**) Latvian dark-head breed lambs. The sequence of multi-locus genotypes is shown using standard IUB/IUPAC nucleic acid codes; “Rear” refers to the group of rare genotypes TTACTA, TTGTTG, KTRYKR, TTACKR, TTAYTR, TTRYTR and TCATTG. X—average value; value in tinted box—median (middle line for a box); cut-off from the minimum to the maximum value; points outside the cut-off—outside the limit value; *p*—the statistical significance.

In analysing multi-loci genotypes across all samples (Figure 2A), the highest slaughter yield, 47.10 ± 1.07%, was determined in samples with the KYRTGG genotype. In contrast, the lowest, 42.88 ± 1.39%, was found in samples with the KYRTKG genotype.

In the LT group of lambs (Figure 2B), the highest slaughter yield, 46.10 ± 0.42%, was observed in lambs with the KYRTGG genotype. The lowest value, 41.83 ± 1.51%, was associated with the TYAYTR genotype. The genotype with the lowest slaughter yield across all samples also had the second lowest average in the LT group, at 42.88 ± 1.39%.

### 3.3. Genotypes in the Intron 2 Region of MSTN Gene

In the intron 2 region, five variable loci were identified in Latvian sheep breed samples, resulting in a total of 10 genotypes. One SNP, Ms19, was found only in a heterozygous form in a single lamb of the CH breed (Table S1), which also exhibited heterozygosity in a rare locus within the intron 1 region.

After excluding the rare variable locus from the multi-locus genotypes, nine genotypes remained, four of which were rare, each occurring in only one sample. Specifically, one genotype was found in the DOR and CH breeds and two were unique to LT breed lambs.

The most common genotype across all breeds and within the LT group was ATCA, representing homozygous forms of the common alleles at all loci (Figure 1C). This genotype was present in 40.79% of all samples and 29.17% of the LT samples. The second most frequent genotype in both groups was RTYM, a heterozygous form at three loci, occurring in 26.32% of all samples and 27.08% of the LT samples. The distribution of genotypes among samples in the LT group was more uniform compared to the group of all samples.

**Figure 3:**
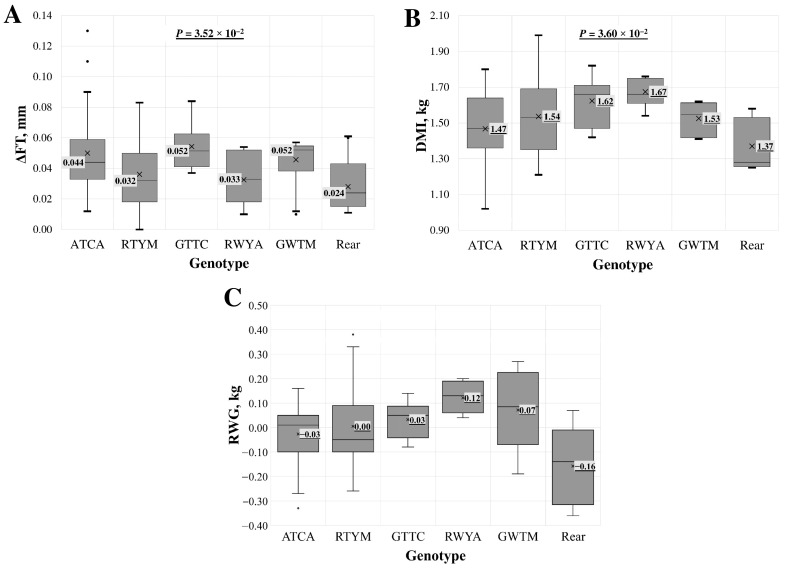
Association of intron 2 region multi-locus genotypes with (**A**) delta of fat thickness (ΔFT) at the 13th rib per kilogramme of gained body weight, (**B**) dry matter intake (DMI), and (**C**) residual weight gain (RWG) in the entire lamb group. Multi-locus genotypes are represented using standard IUB/IUPAC nucleic acid codes. “Rear” refers to the group of rare genotypes: ATYM, GATA, RTYA, and RTCC. X—average value; value in tinted box—median (middle line for a box); cut-off from the minimum to the maximum value; points outside the cut-off—outside the limit value; *p*—the statistical significance.

For intron 2 multi-locus genotypes, a statistically significant association was found across all samples but not when analysing the LT samples separately. The association (*p* < 0.05) was identified with the delta of fat thickness (ΔFT) at the 13th rib per kilogramme of gained body weight, dry matter intake (DMI), and residual weight gain (RWG) (Figure 3). For the last two indicators, the association in the LT group was on the borderline of statistical significance.

The change in fat thickness (ΔFT) during feeding is ideally kept as small as possible, favouring muscle tissue formation over fat accumulation. The smallest ΔFT was observed in the group of rare genotypes (Figure 3A), with a median increase of 0.022 mm. The second smallest increase, 0.032 mm, occurred in the RTYM genotype. The multi-locus genotype GTTC, featuring the rare allele homozygous at three loci, and the GWTM genotype showed the greatest increase in fat thickness at the 13th rib, with an increase of 0.052 mm.

In the single-locus analysis, an association with ΔFT was identified for the SNPs c.747+164A>G (rs426500486) and c748-810C>T (rs423466211) (Table S2). In both loci, samples with the heterozygous form had the lowest ΔFT, at 0.031 mm, while the highest ΔFT was observed in lambs with the rare allele homozygous. This relationship was also confirmed in the multi-locus analysis, where the highest ΔFT was found in genotypes with GG at rs426500486 and TT at rs423466211, and the lowest ΔFT was found in heterozygous cases.

The lowest dry matter intake (DMI), at 1.40 ± 0.16 kg (Figure 3B), was observed in lambs with rare genotypes, followed by 1.47 ± 0.21 kg in lambs with the ATCA multi-locus genotype. The highest DMI, 1.67 ± 0.08 kg, was found in lambs with the RWYA multi-locus genotype.

In the LT lamb group, intron 2 genotypes showed a borderline statistically significant association with DMI (*p* = 0.053) and no significant association at the single-locus level.

In the analysis of feed efficiency indicators, a higher RWG is more desirable (Figure 3C). The highest RWG, 0.121 ± 0.06 kg, was found in lambs with the RWYA multi-locus genotype. The lowest RWG, −0.108 ± 0.14 kg, was observed in lambs with rare genotypes, followed by the second lowest RWG, −0.027 ± 0.13 kg, in lambs with the ATCA genotype. No single locus was associated with RWG in this analysis [14].

### 3.4. Genotypes in the 3′UTR Region of the MSTN Gene

Four variable loci were identified in the 3′UTR in Latvian lamb samples. Two loci (rs414527527 (c.*709A>C) and rs419982449 (c.*1316G>A)) were found only in a single lamb from the CH breed, exhibiting heterozygosity in other regions. After excluding these two loci from the analysis, six genotypes were identified, three of which were considered rare (Figure 1D). The c.*1232G>A (rs408469734) locus, included in the 3′UTR multi-locus genotypes, was homozygous in Latvian dark-head samples. Consequently, only three genotypes were determined in the LT group, one of which was rare.

The most frequent genotype, accounting for 63.16% of all samples, was TG, representing the homozygous genotype of the common allele at both loci. This specific genotype was present in 68.75% of LT lambs.

In the single-locus analysis (Table S2), SNP rs591795591 (c.*707DelT) was found to be in complete linkage disequilibrium with rs119102826 and rs408710650 [14]. In the LT samples, the association between the specific SNPs group and the delta of muscle depth (ΔMD) at the 13th rib per kilogramme of gained body weight, as well as the delta of the MD/FT ratio, was on the borderline of statistical significance. However, no statistically significant associations were identified with any of the analysed characteristics in the samples from all breeds.

For c.*1232G>A, an association was found in samples from all breeds with various traits, including birth weight, DMI, ADG, ΔFT, slaughter yield, muscle development, fat degree, RGR, and KR. Lambs with the homozygous GG genotype for the common allele had, on average, a 0.25–0.28 kg higher DMI, a 77.39–82.42 g higher ADG, a 0.12% per day higher RGR, a 3.6–4.25 points higher KR, and 0.003 mm less ΔFT. Conversely, lambs with the rare homozygous AA genotype showed an average birth weight that was 0.86 to 1.42 kg higher and a slaughter yield that was approximately 4% higher than those of GG genotype lambs. Additionally, muscle development and fat degree evaluations were either better or lower in AA genotype lambs.

By combining the c.*707DelT and c.*1232G>A variants, a statistically significant difference between genotypes was identified in the all-sample group of lambs for several previously established characteristics (Table 1), including birth weight, DMI, ADG, slaughter yield, muscle evaluation, fat degree, and RGR, as well as a previously unreported RWG. The relationships observed were as follows: for birth weight, slaughter yield, muscle development, and fat degree, the order was TA > Rear > NG ~ TG; for the other indicators, the order was NG ~ TG > Rear > TA.

Lambs with the TA genotype had a higher birth weight, averaging 5.76 ± 0.57 kg, while the average birth weight for lambs with other genotypes was less than 5.00 kg. The TA genotype also showed better results in slaughter yield, with an average of 48.76 ± 2.08%, compared to 44.46 ± 2.30% and 44.92 ± 2.66% for lambs with the TG and NG genotypes, respectively.

The median scores for muscle development and fat degree were 2.00 and 2.50 points, respectively, for lambs with the TA genotype, while both indicators were 3.00 for lambs with the NG and TG genotypes. Lambs with the NG genotype consumed an average of 1.55 ± 0.16 kg of concentrate and gained 0.35 ± 0.07 kg daily. In contrast, lambs with the TA genotype had the lowest values, with a DMI of 1.27 ± 0.18 kg and an ADG of 0.26 ± 0.03 kg. Lambs with the TG genotype consumed an average of 1.54 ± 0.19 kg of concentrate and gained 0.33 ± 0.07 kg per day.

Despite the DMI and ADG being statistically significantly different among lambs with different 3′UTR region genotypes, no significant difference was found in the FCR. However, the results showed that lambs with the NG genotype had an FCR of 4.99 ± 0.96 kg, while those with the TA genotype had an FCR of 5.52 ± 1.21 kg. Lambs with the TG genotype had an average FCR of 5.07 ± 0.99 kg.

Lambs with the NG and TG genotypes had similar RGR values, at 0.37 ± 0.08% and 0.35 ± 0.10% per day, respectively, and similar KR medians, at 19.13 and 18.67 points. In contrast, lambs with the TA genotype had significantly lower values for both indicators, with an RGR of 0.24 ± 0.02% per day and a KR of 15.72 points.

## 4. Discussion

In Latvia, sheep populations have decreased over the past decade and the demand for high-quality sheep meat has increased [35]. In the territory of Latvia, there are ten sheep breeds with breeding programmes, but only one national breed—the Latvian dark-head sheep. This breed is fully adapted to Latvia’s climatic conditions [41]. Therefore, preserving and improving this breed has been a priority not only for sheep breeders but on a national level.

Maintaining pure bloodlines in the breeding process is crucial; therefore, an alternative method, such as interbreeding between different breeds, is needed to select for various traits. Marker-assisted selection can be employed for this purpose, using molecular DNA markers identified in scientific studies of the specific breed [18,32]. However, studies searching for molecular markers should focus on preferred and comparable cultivars with superior performance to identify desirable trait loci.

Latvian sheep breeding has ten breeding programmes [21] wherein breed development is ensured by preserving bloodlines and using phenotypic indicators. For the last ten years, genetic analysis or MAS, involving three variations of one gene, has been performed on sheep breeds in Latvia to improve sheep resistance to scrapie disease [33]. Applying molecular research results to practical sheep farming has resulted in a sheep population free of scrapie outbreaks.

Similar positive experiences using MAS and estimated breeding values (EBVs) to rule out scrapie have also been found in other sheep populations [42]. Thus, the results obtained in a particular study in Latvia can be used to develop MAS to improve fattening, feed efficiency, and carcass traits by calculating EBVs in the future.

The marker-assisted selection offers a valuable tool for improving desirable traits. MAS relies on previously established relationships between DNA loci and/or multi-loci combinations and specific characteristics, forming quantitative trait loci (QTL) within a breed [32]. The SheepQTLdb database (https://bioregistry.io/registry/sheepqtldb; accessed on 25 July 2024) contains information about QTL in various sheep breeds, but there is no information about the Latvian dark-head. In addition to using a specific QTL in the MAS process, the conditions in which the research has been carried out, or the region, are of great importance. Thus, it is important to analyse not only different breeds but also similar breeds in different regions.

Our study examined the LT breed and other breeds commonly raised in Latvia, allowing us to investigate the genetic variability of sheep breeds in the region. The results of MSTN gene variations confirmed that particular loci are variable only in specific breeds. A similar conclusion was reached by Han and colleagues [10] in their study of the complete MSTN gene in the New Zealand sheep population. When comparing the polymorphisms in Latvian sheep populations with those in other regions [4,8,9,10,11,12,13], differences were observed between breeds and within populations of the same breed in different regions.

Intron 1 of the MSTN gene and its SNP cluster are among the most frequently genotyped regions across different breeds. Many studies have determined the haplotypes of variable SNPs, and association analyses have been conducted at both the single-locus level and by creating multi-locus genotypes [1,8,9,12,13]. In one study, the analysis of multi-locus genotypes involving three SNPs—rs119102826, rs427811339 and rs119102828—and revealed a relationship between heart circumference and leg circumference in the Iranian Makuei sheep breed [19].

In the Coloured Polish Merino breed ram lamb’s population [12], an analysis of seven SNPs in multi-locus genotypes revealed associations with chest depth, fat depth over the ribs at the thickest point, and various slaughter measurements, including slaughter yield. In contrast, in the Polish Kamieniec sheep breed, the same seven SNPs of the MSTN gene in multi-locus genotypes were associated with body weight at 56 days and average daily gain (ADG) [13].

Our findings confirm the association between intron 1 region multi-locus genotypes and slaughter yield. Combined with the results of other studies, this suggests that MSTN gene intron 1 multi-locus genotypes are associated with slaughter yield not just in specific breeds but potentially across the entire sheep population.

The results of our study highlight the significance of rs119102828 (c.373+259G>T) SNP in the observed association. We identified a relationship with this specific SNP in the single-locus analysis. Moreover, when the SNP alleles varied within multi-locus genotypes, the impact on slaughter yield was more pronounced than in the single-locus analysis. Similarly, in the Coloured Polish Merino breed [12], rs119102828 was one of the critical SNPs in the multi-locus genotypes associated with significant lambs’ biometric traits and carcass and meat quality, including pre-slaughter weight and intramuscular fat content.

The MSTN gene intron 2 region has rarely been analysed. Our study identified a relationship between MSTN intron 2 multi-locus genotypes and ΔFT, DMI, and RWG across six sheep breeds in Latvia without distinguishing specific breeds. The results for DMI and RWG suggest that the RWYA multi-locus genotype is preferable for feed efficiency because it is associated with more significant weight gain while consuming less feed—over 0.200 kg more per day—and shorter durations to achieve this gain. Additionally, lambs with this genotype exhibit a lower ΔFT, indicating that muscle, rather than fat, is being developed. However, it is essential to note that the prevalence of this genotype in the population is below 10%.

Lastly, the 3′UTR region of the MSTN gene was examined. Four polymorphisms were detected in Latvian sheep samples, though the literature [10] describes at least five additional loci that were non-variable in Latvian samples. Our single-locus analysis highlights the significance of rs408469734 (c.*1232G>A), which was not variable in LT breed lambs, and its variability in other breeds was consistent with findings from previous studies [8,10,12,13,14,21,22,43].

Previous research has shown that the SNP rs408469734 (c.*1232G>A) is associated with body frame and/or growth characteristics in various breeds [13,21], the same as for sheep breeds in Latvia. However, it is essential to note that the individual significance of c.*1232G>A has been the primary focus of the above studies.

Studies have found the c.*1232G>A SNP to be associated with carcass and meat quality traits across different breeds [13,21,22,23]. For instance, an analysis of British Charollais lambs [22] identified an association between c.*1232G>A and muscle depth at around 22 weeks of age at the third lumbar vertebra. In the Polish Kamieniec sheep breed, c.*1232G>A SNP genotypes were linked to body weight at 56 days and through ADG, with the heterozygous genotype showing better performance [13].

For multi-locus genotypes in Latvian sheep, the association is observed for the same indicators as for SNP Ms22 separately. However, when examining the actual indicator values, it becomes clear that the value difference is more pronounced when rs591795591 (c.*707DelT ) SNP genotypes are included in the multi-locus genotypes. This suggests that, while the c.*707DelT SNP is not individually significant, its significance increases when combined with the influential c.*707DelT SNP.

Previous research has shown that the SNP rs408469734 (c.*1232G>A) is located in a region targeted by specific microRNAs, leading to increased levels of MSTN protein in the circulation. As a result, the mutant allele is favoured for enhancing growth characteristics [8,44]. There is no information about a possible association between the c.*707DelT SNP and a miRNA attachment site, as this particular SNP has not been identified in the more commonly analysed breeds. Information about the frequency of c.*707DelT is available only in the Ensemble database about Iranian and Moroccan sheep from the NextGen project.

In the study, three SNPs in the coding part were found, one of which, c.84G>A, was previously unspecified in the database. All three SNPs in Latvia were found in only one breed: exon 1 SNPs c.85G>A and rs417816017 in the MLS breed and exon 2 SNP rs410961001 in the DOR breed. The other SNPs in the coding part from the Ensemble database, or which were described in publications on sheep breeds in Latvia, were monozygotic, re-proving the conservatism of the coding part and differences between varieties.

Collecting data from the database, previous publications [17,18] and our study, 12 variations in the coding part of the MSTN gene have been found in different breeds.

This study establishes an association between MSTN gene multi-locus genotypes and fattening indicators. This suggests these genotypes can serve as genetic markers for marker-assisted selection to improve Latvian sheep, particularly the national Latvian dark-head breed. Specifically, there is a substantial market demand for lamb meat, particularly for lambs aged 6–8 months and weighing between 40 and 55 kg before slaughter.

The MAS can be strategically employed in two ways [45]. Firstly, it can assist in selecting pure-bred offspring with desired genetic DNA profiles and estimated breeding values (EBVs) for traits, optimising sheep crossing outcomes. Second, analysing the genetic markers of newborn lambs enables predicting their productive potential, aiding in decisions about their inclusion in breeding programmes. Accordingly, when introducing MAS in the breeding programme, it is possible to use both ways at the beginning: testing ewes and rams before crossing and testing newborns. In time, it is possible to switch to genotyping only newborn lambs, as information on the population will be accumulated, and the desired breeding animals, ewes and rams, can be selected.

However, it should be mentioned that gene polymorphisms, especially multi-locus genotypes, are not as common as molecular markers in sheep breeding. However, some studies integrate genomic DNA variation in determining EBV. Carracelas et al. [46] and Kasej et al. [47] have investigated the best linear unbiased prediction (BLUP) models using DNA variation derived from genotyping large numbers of SNPs. Such processes do not analyse the genotypes of single-gene variations.

In addition, EBV detection models are often designed for specific external conditions, specific nutrition, and specific regional conditions [48]. This indicates the need to study not only different breeds but also to concentrate and use uncontrolled conditions in genetic marker analyses in the future.

However, it is crucial to recognise that further research is needed before implementing MAS. This includes increasing the population of genotyped lambs by incorporating more samples and more representatives from each breed. Doing so would allow for a more comprehensive analysis of the association between full gene multi-locus genotypes and various indicators. In addition, increasing the number of samples would make the detection of identical genotypes in several breeds more likely. This would allow for the comparison of lambs of different breeds with identical genotypes, helping to determine the influence of both the MSTN gene and the breed on these traits.

Our study also highlights the importance of considering opposing genotypes. Therefore, in the MAS process, it is crucial to balance selecting one trait over another, ensuring that the selection for one genotype does not inadvertently diminish the value of another important trait.

## 5. Conclusions

Our analysis of MSTN gene polymorphisms in the Latvian sheep population reveals that MSTN gene polymorphism variability depends on the breed. This underscores the importance of studying different sheep breeds in Latvia, particularly the national Latvian dark-head breed.

Among the genotypes analysed, lambs with the MSTN gene intron 1 region multi-locus genotype KYRTGG had the highest slaughter yield. In the overall Latvian pure-breed sheep population, and within the Latvian dark-head breed group, lambs with the KYRTKG genotype had the lowest slaughter yield.

The RWYA genotype of the MSTN gene intron 2 region is preferred in the overall Latvian pure-breed sheep population. Lambs with this multi-locus genotype gain more weight while consuming less feed, resulting in a shorter fattening duration. Additionally, these lambs exhibit a more minor increase in fat thickness at the 13th rib per kilogramme of weight gained during the fattening period.

For traits, birth weight, slaughter yield, muscle development, and fat degree in the overall Latvian pure-breed sheep population in regard to the 3′UTR loci c.*707DelT/c.*1232G>A is the preferred TA genotype, but for dry matter intake, average daily gain, relative growth rate, and residual weight gain, the NG and TG genotypes are preferred.

Our results indicate that the MSTN gene plays a significant role in intensive lamb fattening, and incorporating these findings into breeding practises could enhance sheep breeds in Latvia and improve economic returns.

## Figures and Tables

**Table 1:** A statistically significant difference was found between 3′UTR region multi-locus genotype groups for various parameters in the all-sample group of sheep breeds in Latvia.

Parameter		Genotype of 3′UTR (c.*707DelT/c.*1232G>A)				*p*
		T/G	N/G	T/A	Rear *	
Birth weight, kg	Mean ± SD	4.34 ± 0.81	4.32 ± 0.56	**5.76 ± 0.57**	4.85 ± 0.13	4.76 × 10^−4^
	Median (IQR)	4.30 (1.15)	4.20 (1.00)	5.60 (1.00)	4.85 (0.25)	-
Average daily gain, g	Mean ± SD	333.35 ± 66.88	349.86 ± 66.88	261.30 ± 34.68	286.97 ± 96.57	-
	Median (IQR)	321.74 (109.44)	**352.00 (82.61)**	272.73 (75.32)	321.33 (174.28)	3.72 × 10^−2^
Dry matter intake, kg	Mean ± SD	*1.54 ± 0.19*	1.55 ± 0.16	**1.27 ± 0.18**	1.37 ± 0.28	2.35 × 10^−3^
	Median (IQR)	1.53 (0.27)	1.53 (0.26)	1.24 (0.34)	1.42 (0.53)	-
Slaughter yield, %	Mean ± SD	44.64 ± 2.30	44.92 ± 2.66	**48.76 ± 2.08**	46.55 ± 2.64	2.29 × 10^−3^
	Median (IQR)	44.15 (3.38)	44.90 (4.40)	49.4 (3.40)	46.05 (4.80)	-
Muscle development	Mean ± SD	2.84 ± 0.42	2.89 ± 0.27	**1.90 ± 0.55**	2.38 ± 0.75	-
	Median (IQR)	3.00 (0.00)	3.00 (0.00)	2.00 (0.75)	2.50 (1.38)	1.00 × 10^−4^
Fat degree	Mean ± SD	2.95 ± 0.38	2.97 ± 0.61	**2.40 ± 0.22**	2.50 ± 0.58	-
	Median (IQR)	3.00 (0.00)	3.00 (0.50)	2.50 (0.25)	2.50 (1.00)	3.70 × 10^−3^
RGR	Mean ± SD	0.35 ± 0.10	**0.37 ± 0.08**	0.24 ± 0.02	0.26 ± 0.09	2.02 × 10^−2^
	Median (IQR)	0.33 (0.14)	0.38 (0.13)	0.25 (0.04)	0.28 (0.16)	-
KR	Mean ± SD	18.50 ± 2.88	**18.83 ± 2.58**	15.02 ± 1.26	15.79 ± 4.78	-
	Median (IQR)	18.67 (3.75)	19.13 (3.94)	15.72 (2.39)	17.05 (8.54)	2.03 × 10^−2^
RWG	Mean ± SD	**0.02 ± 0.14**	−0.01 ± 0.12	−0.14 ± 0.20	−0.06 ± 0.17	9.94 × 10^−3^
	Median (IQR)	0.04 (0.18)	−0.03 (0.17)	−0.26 (0.37)	−0.03 (0.31)	-

The sequence of multi-loci genotypes is represented in the standard IUB/IUPAC nucleic acid codes with one letter for each locus. * Rear genotype: T/R, -/G, N/R; in bold—the desired or best result; underlined—the worst result. *p*—statistical significance using the ANOVA test for normally distributed data (in the table against the mean) or the Kruskal–Wallis test for non-normally distributed data (in the table against the median).

## Data Availability

Data on feed efficiency indicators of LT lambs can be found in Trapina Ilva. (2023). The database of feed efficiency indicators of intensive fattening lambs of Latvian sheep breeds within the framework of the Latvian Council of Science LZP-2021/1-0489 project [Data set]. Zenodo. https://doi.org/10.5281/zenodo.8143244.

## Supplementary Materials

The following supporting information can be downloaded at: https://www.mdpi.com/article/10.3390/ani14213143/s1, Table S1. The variable MSTN gene polymorphism variability in breeds and haplotype analysis in the Latvian sheep population. Table S2. MSTN gene common SNPs single loci association analysis in the Latvian sheep population and Latvian dark-head group.
