# Supplementary material for: The Multi-Loci Genotypes of the Myostatin Gene Associated with Growth Indicators of Intensively Fattened Lambs of Latvian Sheep
Source: Animals (Basel). 2024 Nov 1;14(21):3143. doi: 10.3390/ani14213143 (PMC11545172; doi:10.3390/ani14213143)
Supplement: Supplementary file 1 [file animals-14-03143-s001.zip › animals-3255979-supplementary.pdf]

Table S1. Variable MSTN gene polymorphisms variability in breeds and haplotype analysis in Latvian sheep population.

	Promoter		Exon 1		Intron 1									Ex2	Intron 2					3'UTR							
	rs411139795	rs119102824	New_1	rs417816017	rs119102825	rs119102826	rs427811339	rs406172342	rs417602601	rs119102828	rs408710650	rs419902890	rs399737483	rs410961001	rs426500486	rs404916326	rs423466211	rs406265773	rs428638621	rs591795591	rs414527527	rs408469734	rs419982449				
	c.-40C>A	c.-37T>C	c.84G>A	c.101A>G	c.373+18G>T	c.373+241T>C	c.373+243G>A	c.373+246T>C	c.373+249T>C	c.373+259G>T	c.373+563G>A	c.373+607G>A	c.374-54C>T	c.384A>G	c.747+164A>G	c.747+309T>A	c748-810C>T	c.748-475A>C	c.748-54C>T	c.*707DelT	c.*709A>C	c.*1232G>A	c.*1316G>A				
Breed	C>A	C>T	G>A	A>G	G>T	T>C	G>A	T>C	T>C	G>T	G>A	G>A	C>T	A>G	A>G	T>A	C>T	A>C	C>T	DelT (T>-)	A>C	G>A	G>A				
LT	X	X			X	X	X	X		X	X	X	X		X	X	X	X		X							
IF	X	X			X	X	X	X		X	X	X	X		X		X	X		X							
TEX	X				X	X	X				X				X		X	X		X		X					
CH	X	X			X		X	X	X	X		X	X		X		X	X	X		X	X	X				
DOR	X	X			X		X	X		X		X	X	X	X		X	X									
MLS	X		X	X	X	X	X				X				X		X	X		X							
Haplotypes																								All (152)		LT (96)	
H1	C	T	G	A	G	T	G	T	t	G	g	G	c	a	A	T	C	A	c	t	a	G	g	68	44.74	49	51.04
H2	A	T	G	A	T	C	A	T	t	T	a	G	c	a	G	T	T	C	c	c	a	G	g	15	9.87	14	14.58
H3	A	T	G	A	T	T	G	T	t	T	g	G	c	a	G	A	T	A	c	t	a	G	g	15	9.87	15	15.63
H4/5	A	C	G	A	T	T	A	C	t/c	T	g	A	t	a	G	T	T	C	c/t	t	c/a	G	g/a	13+1	9.21	8	8.33
H6	C	T	G	A	G	T	G	T	t	G	g	G	c	a	A	T	C	A	c	t	a	A	g	12	7.89	0	0.00
H7	A	T	G	A	T	C	A	T	t	G	a	G	c	a	G	T	T	C	c	c	a	G	g	7	4.61	2	2.08
H8	A	C	G	A	T	T	A	C	t	G	g	A	t	a	G	T	T	C	c	t	a	G	g	6	3.95	5	5.21
H9	A	T	G	A	G	T	G	T	t	G	g	G	c	a	A	T	C	A	c	t	a	G	g	6	3.95	2	2.08
H10	C	T	G	G	G	T	G	T	t	G	g	G	c	a	A	T	C	A	c	t	a	G	g	2	1.32	0	0.00
H11	C	T	A	A	G	T	G	T	t	G	g	G	c	a	A	T	C	A	c	t	a	G	g	2	1.32	0	0.00
H12	A	C	G	A	T	T	A	C	t	G	g	A	t	g	A	T	T	C	c	t	a	G	g	1	0.66	0	0.00
H13	A	T	G	A	T	T	G	T	t	T	g	G	c	a	G	T	T	A	c	t	a	G	g	1	0.66	1	1.04
H14	C	T	G	A	T	T	A	C	t	G	g	G	c	a	A	T	C	C	c	t	a	A	g	1	0.66	0	0.00
H15	C	T	G	A	T	T	A	C	t	T	g	A	t	a	G	T	C	C	c	t	a	G	g	1	0.66	0	0.00
H16	A	T	G	A	T	T	A	T	t	T	g	G	c	a	A	T	C	A	c	t	a	G	g	1	0.66	0	0.00

LT: Latvian dark-head (Latvijas tumšgalve); MLS; Merinolandschaf, IF: Île de France; CH: Charollais; DOR: Dorper; TEX: Texel; All – all samples of all breeds; different colouring for different linkage disequilibrium groups; small letters for SNPs not included in multi-loci genotypes analyses.

Table S2. MSTN gene common SNPs single loci association analysis in the Latvian sheep population and Latvian dark-head group.

Region	Polymorphisms			Indicators								
				DMI	AGD	BWbirth	BW90th	BW150th	ΔMD	ΔFT	Δ(MD/FT)	SW %
Pro-moter	rs411139795	C>A	All	*CC<CA<AA*								
			LT									
	rs119102824	C>T	All									
			LT				CC>CT>TT					
Exon 1	New_R	G>A	All									
			LT	Monozygotic or not variable								
	rs417816017	A>G	All									
			LT	Monozygotic or not variable								
Intron 1	rs119102825	G>T	All	**GG<TT<GT**		TT>GT>GG				**GT<GG<TT**	**GT>GG>TT**	
			LT				TT>GT>GG	GT>TT>GG	**GG>TT>GT**	GT<TT<GG		
	rs119102826	T>C	All									
			LT						TT>TC			
	rs427811339	G>A	All									
			LT				AA~GA>GG					**GA>GG>AA**
	rs406172342	T>C	All									
			LT				CC>TC>CC					
	rs119102828	G>T	All			**GT>TT>GG**				GT<GG<TT	GT>GG>TT	**GG>TT>GT**
			LT									
	rs408710650	G>A	All									
			LT						GG>GA			
	rs419902890	G>A	All									
			LT				AA>GA>GG					
Intron 2	rs426500486	A>G	All	AA<AG<GG		**AG>GG>AA**				**AG<AA<GG**	**AG>AA>GG**	
			LT				GG>AG>AA	AG>GG>AA	**AA>GG>AG**	AG<GG<AA		
	rs404916326	T>A	All	**TT<AA<TA**								
			LT	**TT<AA<TA**								
	rs423466211	C>T	All	**CC<CT<TT**		**CT~TT>CC**				**CT<CC<TT**	**CT<CC<TT**	
			LT				TT>CT>CC	CT>TT>CC	**CC>TT>CT**	CT<TT<CC		
	rs406265773	A>C	All									
			LT				AC>CC>AA					
3′UTR	rs591795591	DelT (T>-)	All									
			LT						TT>T-			
	rs408469734	G>A	All	**AA~GA<GG**	**GG>AA>GA**	**AA<GA<GG**				**GA<GG<AA**		**AA<GA<GG**
			LT	Monozygotic or not variable								

All – all samples of all breeds; LT - Latvian dark-head group; DMI – dry matter intake; ADG - average daily gain; BW – body weight at birth, at 90th and 150th day; ΔMD and ΔFT - the change (delta) of muscle depth and fat thickness of the 13th rib per one kg gained body weight in time of fattening; SW % - slaughter weight in % from the live weight of each lamb at the time of processing; In bolds – statistically significant (P < 0,05) single locus genotype relationship; underlined - single locus genotype relationship on boarder of statistical signification P = [0.05 – 0.09]
